# Thiol-Ene Coupling of High Oleic Sunflower Oil towards Application in the Modification of Flexible Polyurethane Foams

**DOI:** 10.3390/ma15020628

**Published:** 2022-01-14

**Authors:** Sylwia Dworakowska, Adrien Cornille, Dariusz Bogdal, Bernard Boutevin, Sylvain Caillol

**Affiliations:** 1 Faculty of Chemical Engineering and Technology, Cracow University of Technology, Warszawska 24, 31-155 Cracow, Poland; dariusz.bogdal@pk.edu.pl; 2 ICGM, University of Montpellier, CNRS, ENSCM, 34000 Montpellier, France; adrien.cornille@enscm.fr (A.C.); bernard.boutevin@enscm.fr (B.B.)

**Keywords:** bio-based polymers, polyurethanes, flexible foam, vegetable oil, thiol-ene coupling

## Abstract

High oleic sunflower oil-based polyol was obtained by thiol-ene coupling and applied in the preparation of flexible polyurethane foams. The photochemically initiated thiol-ene click reaction was carried out under UV irradiation using 2-mercaptoethanol. Bio-based polyol with hydroxyl value of 201.4 mg KOH/g was used as 30 wt% substituent of petrochemical polyether polyol in the formulations of flexible foams. Both reference foams, as well as foams modified with bio-based polyol, were formulated to have various isocyanate indices (0.85, 0.95, 1.05). Flexible foams were compared in terms of their thermomechanical properties and analyzed using FT-IR and SEM microscopy. Modification with bio-based polyol resulted in foams with superior compression properties, higher support factor, and lower resilience than reference foams. TGA and FT-IR curves confirmed the presence of urethane/urea and ether linkages in the polyurethane matrix. Moreover, double glass transition temperature corresponding to soft and hard segments of polyurethane was observed by DSC proving the phase-separated morphology.

## 1. Introduction

Development of bio-based polymers is connected with looking for green, low cost and environmentally friendly raw materials. Nowadays, polymers derived from renewable sources are gaining great interest both in academia and industry. Following the concept of sustainable development, scientific research is focused on finding new bio-based monomers to replace petroleum-based ones [1,2,3,4]. It can be achieved by e.g., thiol-ene addition (or thiol-ene click reaction) of thiols to the double bonds of organic compounds. This click-chemistry reaction [5] follows the Green Chemistry principles [6,7] and is particularly important in obtaining bio-based monomers since numerous renewable sources, such as vegetable oils or terpenes, have C=C double bonds [8].

The addition of thiols to unsaturated compounds was discovered in 1905 by Posner [9] and applied for the first time in 1948 [10]. This reaction proceeds in an anti-Markovnikov manner using metal-catalyzed complexation [11] or free radical mechanism [12,13,14] and can be initiated photochemically by ultraviolet (UV) light (wavelength 200–400 nm) and photoinitiators, or thermally in the presence of radical initiators. Thiol-ene reaction is carried out under mild conditions and characterized with high yield, high reaction rates, and absence of byproducts if an equimolecular ratio of thiol groups to double bonds is used [12,15,16,17].

The radical addition of thiols to fatty acids and their esters received increased interest recently and was by now broadly described in the literature [18,19,20,21,22,23,24,25]. This one-step method represents a significant advance in comparison to the traditional two-step epoxidation and oxirane ring-opening approach. Previously, we synthesized bio-based polyol by thiol-ene coupling of vegetable oil with 2-mercaptoethanol and afterward polymerized it using various diisocyanates to obtain polyurethane elastomers [24,25]. The best results were achieved when the thiol-ene reaction was carried out at 3:1 thiol to diene molar ratio, without solvent, and under UV irradiation. Therefore, we decided to apply these conditions in thiol-ene modification of vegetable oil to obtain polyol and subsequently use it in the synthesis of polyurethane foams. Typically, bio-based polyurethane foams are used in the applications such as mattresses, furniture, insulation, and automotive (e.g., seating systems, interior parts, headrests, headliners, and trim covers) [26,27]. Despite extensive research on bio-based polyurethanes [28,29,30,31,32,33,34], as far as we know, there has been no study about the modification of polyurethane foams using bio-based polyol coming from the thiol-ene reaction. Therefore, this study aims to explore the effect of partial substitution of petrochemical polyol with vegetable oil-based thiol-functionalized polyol in the formulation of polyurethane foams on their cell morphology and thermomechanical properties.

## 2. Materials and Methods

High oleic sunflower oil (HOSO, IV = 84.9 g I_2_/100 g) was purchased from Bio Planéte (France). Additionally, 2-Mercaptoethanol (>98%), ethyl acetate (99.5%), potassium hydroxide (KOH, 99.99%), heptane (99%) and methanol (99.9%) were purchased from Sigma-Aldrich and used as received. Rokopol M5000 (trifunctional polyether polyol based on glycerine, HV = 35.8 mg KOH/g, M = 4800 g/mol, f = 3.0, η at 25 °C = 844 mPa·s) was supplied by PCC Rokita S.A. (Poland). TDI 80 (toluene diisocyanate 80/20) was supplied by ZACHEM S.A. (Poland). DABCO DC 5950 surfactant (polysiloxane-polyoxyalkylene copolymer), blow catalysts: DABCO BL-11 (70 wt% N,N,N′,N′-tetramethyl-2,2′-oxy-bis(ethylamine), 30 wt% oxydipropanol), DABCO NE300 (≥90 wt% N-[2-[2-(dimethylamino)ethoxy]ethyl]-N-methyl-1,3-propanediamine), gel catalysts: DABCO 33-LV (67 wt% oxydipropanol, 30 wt% 1,4-diazobicyclooctane), DABCO NE1070 (30–70 wt% 3-dimethylaminopropyl urea) and surface cure catalyst POLYCAT 15 (N′-[3-(dimethylamino) propyl]-N,N-dimethylpropane-1,3-diamine) were supplied by Air Products and Chemicals Inc. Deuterated chloroform (CDCl_3_) was purchased from Eurisotop (Saint-Aubin, France).

^1^H NMR spectra were recorded at 25 °C using a Bruker Avance 400 MHz spectrometer equipped with a QNP z-gradient probe. The chemical shifts for protons are reported in parts per million downfield from tetramethylsilane and are relative to the residual solvent peak used as an internal standard (CDCl_3_: δ = 7.26 ppm).

The fatty acid content in HOSO was tested using an Agilent 6890 N gas chromatograph coupled with a flame ionization detector (GC-FID) after prior derivatization to obtain fatty acid methyl esters. Before injection, the sample of oil (1 µL) was dissolved in 1 cm^3^ of heptane and then 200 µL of 2 M KOH in methanol was added. The operating temperature of detector was 300 °C, the flow of H_2_ 35 cm^3^/min, and the flow of air 400 cm^3^/min. The analytes were separated by capillary column SPTM-2560 with a length of 100 m and diameter of 0.25 mm, coated with bis-cyanopropyl polysiloxane phase (film thickness of this phase was equal to 0.2 µm). The analysis was performed at a constant furnace temperature equal to 180 °C.

The water content was determined by the Karl Fischer method. The study was carried out in accordance with the Polish Norm PN-81/C-04959 using the AquaMAX KF v2.61 titrator with the serial number 71,996 from GR Scientific Ltd.

The viscosity measurements were performed using HAAKE MARS III rheometer from Thermo Scientific Company.

Iodine value (*IV*) was determined according to PN-87/C-04281 Polish Norm. *IV* was calculated from the formula: $IV=\frac{\left(V_{1}-V_{2}\right)\times C_{Na_{2}S_{2}O_{3}\;}\times 0.1269}{m}\times 100$ (g I_2_/100 g), where: *V*_1_—the volume of 1 M Na_2_S_2_O_3_ used for blank probe titration (cm^3^), *V*_2_—the volume of 1 M Na_2_S_2_O_3_ used for sample titration (cm^3^), $C_{Na_{2}S_{2}O_{3}}$—concentration of Na_2_S_2_O_3_ solution (mol/dm^3^), 0.1269—number of iodine grams relative to 1 cm^3^ of 1 M Na_2_S_2_O_3_, and *m*—the mass of sample (g).

Acid value (*AV*) was determined according to PN-ES ISO 2114 Polish Norm. *AV* was calculated from the formula: $AV=\frac{\left(V_{1}-V_{2}\right)\times C_{KOH}\times 56.11}{m}$ (mg KOH/g), where *V*_1_—the volume of 0.1 M KOH used for blank probe titration (cm^3^), *V*_2_—the volume of 0.1 M KOH used for sample titration (cm^3^), $C_{KOH}$—concentration of KOH solution (mol/dm^3^), and 56.11—molar mass of KOH (g/mol).

Hydroxyl value (*HV*) was determined according to PN-93/C-89052/03 Polish Norm. *HV* was calculated from the formula: $HV=\frac{\left(V_{1}-V_{2}\right)\times C_{KOH}\times 56.11}{m}$ (mg KOH/g), where: *V*_1_—the volume of 0.5 M KOH used for blank probe titration (cm^3^), *V*_2_—the volume of 0.5 M KOH used for sample titration (cm^3^), $C_{KOH}$—concentration of KOH solution (mol/dm^3^), *m*—the mass of sample (g), and 56.11—molar mass of KOH (g/mol).

Fourier-transform infrared spectroscopy (FT-IR) tests were performed using Spectrum 65 FT-IR spectrometer (Perkin Elmer) equipped with MIRacle ATR accessory (diamond/ZnSe crystal). Spectra were recorded in the range of 4000–650 cm^−1^ with the resolution of 4 cm^−1^ by performing 32 scans.

Size exclusion chromatography (SEC) was conducted using PL-GPC 50 apparatus from Polymer Laboratories equipped with one PLgel pre-column (3 µm, 7.5 mm × 50 mm, 100 Å), two PLgel mixed-E columns (3 µm, 7.5 mm × 300 mm, 100 Å), and refractive index detector. Columns (thermostated at 35 °C) were calibrated with polystyrene (PS) standards. SEC analyses were carried out with an eluent flow of 1 mL/min using THF stabilized with butylated hydroxytoluene (BHT) with a small amount of toluene as a flow rate marker.

Synthesized foams were seasoned for two days and subsequent samples were cut from the middle section of each foam, according to the applied ISO standards to measure their properties. The compression properties of the foams (stress-strain characteristics) were measured using Zwick testing machine (model Z005 TH Allround-Line) according to Norm ISO 2439–Method E. Resilience of foams was determined according to Polish Norm PN-EN ISO 8307 in both parallel and perpendicular directions to the foam rise. Apparent density was measured in accordance with EN ISO 845:2006 procedure.

The microstructural characterization of the foams was carried out using Scanning Electron Microscopy (SEM, Hitachi S-4700 with EDS system Noran Vantage) with 35× magnification.

Differential scanning calorimetry (DSC) measurements were performed using a NETZSCH DSC200F3 calorimeter calibrated with indium, *n*-octadecane, and *n*-octane standards, under nitrogen atmosphere. Approximately 15 mg of each sample was placed in a pierced aluminum crucible. The thermal properties were analyzed at 20 °C/min in the range from −100 to 100 °C and the glass transition temperature (*T*_g_) values were measured during the second heating ramp. All the reported temperatures are onset values.

Thermogravimetric analyses (TGA) were carried out on a TGA Q50 (TA instrument) at 10 °C/min under nitrogen flow (60 mL/min). Approximately 10 mg of the sample was placed in an aluminum pan and heated from room temperature to 500 °C.

### 2.1. Thiol-Ene Coupling

The photochemical initiator-free thiol-ene reaction was carried out in a quartz reactor placed in a Rayonet RPR-200 UV reactor equipped with 16 UV-lamps of 35 W each with the wavelength of 254 nm. The mixture consisting of 2-mercaptoethanol and HOSO was stirred under the air. The molar ratio between the thiol function and C=C double bond in HOSO was equal to 3:1. The total reaction mixture was 5 g. Aliquots were periodically withdrawn to monitor monomer conversion by ^1^H NMR (by following the disappearance of vinyl proton signals at 5.40 ppm). The photochemical beam was stopped every 1 h to take aliquots. At the end of the thiol-ene coupling, the reaction mixture was dissolved in ethyl acetate (60 mL) and extracted with water (3 × 60 mL) to remove the excess of 2-mercaptoethanol. The organic phase was dried over anhydrous magnesium sulfate and filtered. Next, solvent was evaporated under reduced pressure (3 × 10^−2^ mbar, 30 °C) to recover a pale yellow liquid. Additionally, the same reaction at 400 g scale was carried out to obtain bio-based polyol which could be used in the modification of flexible polyurethane foams. In the case of the thiol-ene reaction carried out on a small scale (5 g) the completion was reached after 9 h. However, for the reaction done on a bigger scale (400 g) the reaction due to less effective mixing had to be done for 3 days (36 h) to reach completion.

### 2.2. Synthesis of Flexible Polyurethane Foams

Flexible foams were obtained by one-step method from two components: A and B. Polyol premix (component A) was a mixture of petrochemical polyol, vegetable oil-based polyol, surfactant, distilled water, and catalysts, while component B was toluene diisocyanate. Table 1 summarizes the formulations used for obtaining flexible foams: reference (REF) and modified with high oleic sunflower oil-based polyol (PHOS). Foams with the isocyanate index (ratio of the equivalent amount of isocyanate used relative to the theoretical equivalent amount times 100) values of 0.85, 0.95, and 1.05 were obtained. The content of the individual component of the formulation was expressed in parts by weight, per 100 parts by weight of the overall polyol component (php). The amount of each catalyst was varied from 0.07 to 0.27 php while the amounts of polyol, distilled water, and surfactant were fixed at 100, 6.5, and 3.1 php, respectively. The water contained in polyols has been included in the calculations of flexible foam formulations. The amount of TDI (in php) required for the reaction with polyol and water was calculated from the equation $m_{TDI}=\left(\frac{m_{polyol}}{OH\;EW}+\frac{m_{H_{2}O}}{H_{2}O\;EW}\right)\times NCO\;EW\times NCO\;index$. In this formula $m_{polyol}$ and $m_{H_{2}O}$ are amounts of polyol and water, respectively, expressed in php, whereas OH EW, $H_{2}O\;EW$, and NCO EW are equivalent weights of hydroxyl groups, water, and isocyanate groups, respectively (g/eq). For the completion of the reaction, excess TDI (ca. 5 wt%) was used. Hydroxyl equivalent weight was calculated from the equation $OH\;EW=\frac{56,100}{HV}$, where *HV* was the hydroxyl value of polyol. Water equivalent weight was equal to 9 since 1 mol of water reacts with 2 moles of isocyanate groups $\left(H_{2}O\;EW=\frac{MW_{H_{2}O}}{f}=\frac{18}{2}=9\right)$. Isocyanate equivalent weight was calculated from the equation $NCO\;EW=\frac{MW_{NCO}\times 100}{\%NCO}=\frac{42\times 100}{48.2}$, where %NCO is isocyanate groups content in TDI expressed in wt%.

Reference foams (REF-85, REF-95, REF-105) contained 100 wt% of Rokopol M5000 petrochemical polyol, while in the case of modified foams (PHOS-85, PHOS-95, PHOS-105) 30 wt% of petrochemical polyol was replaced by bio-based polyol PHOSO. To ensure similar apparent density values of the foams, the same amount of water and surfactant (DABCO DC5950), and various amounts of catalysts were used (selected experimentally depending on the reactivity of polyols). In the formulations, both gelling (DABCO NE1070, DABCO 33-LV) and blowing (DABCO NE300, DABCO BL-11) catalysts, as well as POLYCAT 15 catalyst (accelerating the curing reaction of the foam surface) were used. Among the catalysts were chemicals with reduced amine emissions such as DABCO NE300 and DABCO NE1070.

The synthesis of flexible polyurethane foams was started by mixing the components of the polyol premix using a high-speed mechanical stirrer (2800 rpm) in a one-liter plastic cup. Then, the precisely measured amount of the isocyanate was added. The components were stirred for about 10 s, and then the resulting mixture was poured into an open mold of 16.4 cm × 11.1 cm × 6.5 cm, which allowed for the unobstructed expansion of the reaction mixture in the vertical direction and crosslinking of polyurethane. Flexible foams were obtained at about 20–22 °C and then cured at 80 °C for 15 min. After preparation, the foams were seasoned for two days at room temperature to complete all gelling and blowing reactions and remove unreacted diisocyanate that was used in 5 wt% excess. Subsequently, the foams were cut into the samples, accordingly to the applied ISO standards, and their structural, mechanical, and thermal properties were analyzed as described earlier.

## 3. Results and Discussion

This study is divided into two parts. The first part relates to the synthesis of HOSO-based polyol, while the second one deals with the application of obtained polyol in the preparation of flexible polyurethane foams.

**Figure 1:**
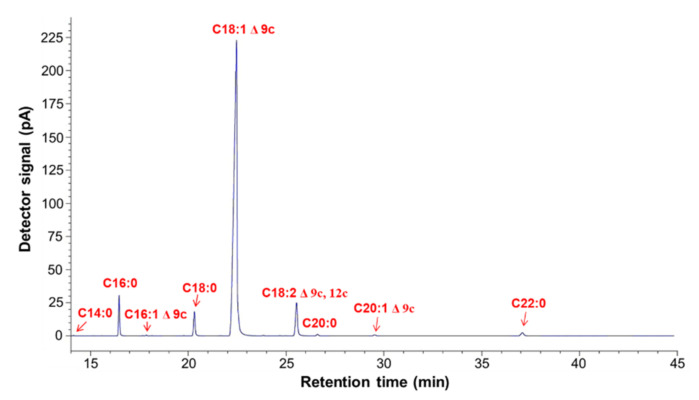
GC-FID chromatogram of HOSO.

First, HOSO was thoroughly characterized before its application in the thiol-ene reaction of bio-based polyol synthesis. *IV* of HOSO determined by titration method was equal to 84.9 g I_2_/100 g (0.335 mol/100 g). The composition of HOSO evaluated by GC-FID revealed the presence of fatty acids in HOSO with the biggest peak being C18:1 Δ 9c, proving its high oleic composition (Figure 1, Table S1). The fatty acids present in HOSO were: C14:0–myristic acid (0.1 mol%), C16:0–palmitic acid (4.1 mol%), C16:1 Δ 9c–palmitoleic acid (0.06 mol%), C18:0–stearic acid (3.3 mol%), C18:1 Δ 9c–oleic acid (85.0 mol%), C18:2 Δ 9c, 12c–linoleic acid (6.0 mol%), C20:0–arachidic acid (0.3 mol%), C20:1 Δ 9c–gadoleic acid (0.3 mol%), and C22:0–behenic acid (0.9 mol%).

Thiol-ene reaction of HOSO was performed under UV irradiation (254 nm) using 2-mercaptoethanol. The product was purified by solvent extraction to remove the remaining amount of thiol. It is important since thiols are susceptible to S–H bond cleavage under UV light, forming disulfides and hydrogen [35].

**Figure 2:**
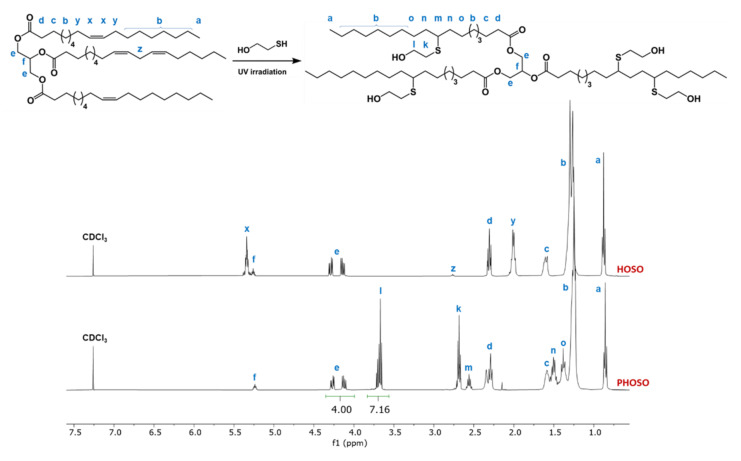
^1^H NMR spectra of HOSO and PHOSO.

First, the reaction of thiol with HOSO at 5 g scale (3:1 thiol:ene molar ratio) was carried out. Afterwards, the same reaction was performed at 400 g scale to obtain polyol that could be used in the preparation of flexible polyurethane foams. The course of the thiol-ene reaction was followed by ^1^H NMR (Figure S1). The signal x corresponding to C**H**=C**H** vinyl protons, centered at 5.33 ppm, shifted slightly downfield to 5.37 ppm after thiol addition (Figure 2 and Figure S1). This shift was a result of *cis*-to-*trans* isomerization of carbon–carbon double bonds. The double-bond isomerization mechanism during thiol-ene coupling reactions has been already studied [36]. The competing *cis*–*trans* equilibrium affects the rate of the final product formation [37]. First, a decrease in the intensity of the signal at 5.33 ppm, corresponding to *cis* double bond, and an increase in the intensity of the signal at 5.37 ppm, corresponding to *trans* double bond, were observed. Next, the intensity of the signal at 5.37 ppm decreased slowly and completely disappeared.

Figure 2 shows ^1^H NMR spectra of HOSO and PHOSO. The spectrum of PHOSO is consistent with the expected product. The quantitative conversion of thiol-ene reaction was monitored by the disappearance of both signals, x at 5.37 ppm and y at 1.98 ppm, assigned to C**H**=C**H** and =CH–C**H**_2_, respectively. A total of 9 h was required to obtain full monomer conversion. The chemical structure of polyol was confirmed by signal m at 2.6 ppm (C**H**–S), signal k at 2.7 ppm (C**H**_2_–S) and signal l at 3.7 ppm (C**H**_2_–OH). The integration of signal l at 3.7 ppm (C**H**_2_–OH) was determined as 7.16 based on the integration of signals e at 4.12–4.28 ppm (–C**H**_2_CHC**H**_2_–) equal to 4.00. This means that one molecule of sunflower oil-based polyol contained approximately three hydroxyl groups.

**Figure 3:**
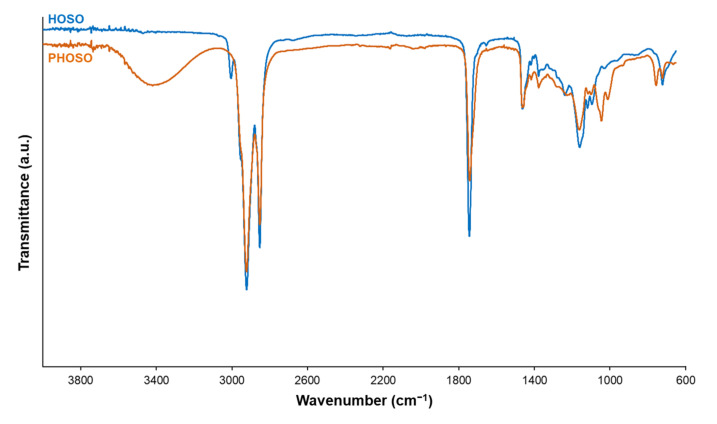
FT-IR spectra of HOSO and PHOSO.

HOSO and PHOSO were compared by FT-IR spectroscopy (Figure 3). The FT-IR spectrum of polyol shows a broad band at 3600–3050 cm^−1^ wavelength which corresponds to the stretching vibrations of hydroxyl groups. Moreover, one can observe the decrease of both the vibration band at 3008 cm^−1^ (stretching C–H sp^2^) and that at 721 cm^−1^ (*cis* C=C deformation), confirming the double bond conversion. The previously described isomerization mechanism during thiol-ene reaction [38,39] can be proved by the presence of C=C bond vibration band at 964 cm^−1^.

**Figure 4:**
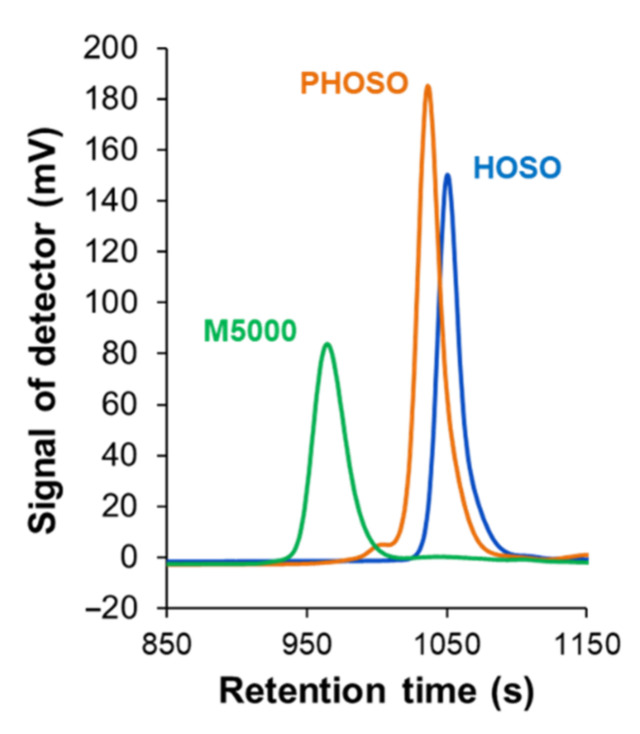
SEC traces of HOSO, PHOSO and M5000.

Table 2 summarizes the properties of HOSO and polyols i.e., high oleic sunflower oil-based polyol (PHOSO) and petrochemical polyether polyol (M5000). Polyols were compared using SEC chromatography to obtain their number average (*M*_n_) and weight average (*M*_w_) molecular weights as well as dispersity (*Ð*) values. SEC chromatograms of HOSO and PHOSO (Figure 4) showed an increase in molecular weight and dispersity, due to thiol addition. Thiol-ene reaction led to 3 wt% of oligomers (dimers and trimers of grafted triglycerides), as shown previously by Bantchev et al. [39]. Oligomerization of vegetable oils has already been studied through thermal and catalytic mechanisms [40] or through self-oxidation mechanism under UV exposure with the formation of hydroperoxides and cyclic peroxides and their subsequent decomposition, yielding radicals that allowed intermolecular coupling [41].

The quantity of free fatty acids, determined by *AV*, was measured to be 0.4 mg KOH/g both before and after thiol-ene coupling reaction. This value showed that hydrolysis was negligible and all the double bonds present in HOSO should be converted into hydroxyls.

*HV* was investigated as 201.4 mg KOH/g for PHOSO and 35.8 mg KOH/g for Rokopol M5000. Polyols exhibited a water content of 0.7648 wt% and 0.5249 wt%, respectively. The functionality of hydroxyl groups present in polyols was calculated based on the equation $f_{OH}=\frac{M_{n}\times HV}{56,110}$ and determined to be 6.14 for PHOSO and 3.00 for Rokopol M5000. Additionally, the viscosity of polyols was evaluated since it is important to ensure appropriate viscosity during the synthesis of foams when the blowing agent is formed. Viscosity at 25 °C was equal to 987.4 mPa·s for PHOSO and 844 mPa·s for Rokopol M5000.

Flexible polyurethane foams were synthesized by reaction of polyol premix with toluene diisocyanate. Both reference foams and modified foams were prepared in accordance with the formulations presented in Table 1. Modified foams were prepared by substitution of 30 wt% of the petrochemical polyol with bio-based polyol obtained by thiol-ene coupling reaction. The petrochemical polyol used in this study, Rokopol M5000, being glycerine-based reactive polyoxyalkylenetriol, is typically used in the production of highly flexible polyurethane foams. Moreover, catalysts responsible for both gelling (forming of urethane linkages) and blowing (evolution of CO_2_ in the reaction of isocyanate with water) reactions were applied. After determination of polyols’ *HV*s, suitable amounts of polyol premix (petrochemical polyol, vegetable oil-based polyol, surfactant, distilled water, and catalysts) and toluene diisocyanate were mixed, poured into the mold, cured at 80 °C for 15 min and then seasoned at 20–22 °C for two days. The properties of the modified flexible foams were compared to the ones based solely on a commercial polyether polyol, Rokopol M5000.

The chemical structure of flexible foams was investigated by FT-IR spectroscopy (Figure S2). For all the foams, N–H vibration bands at 3340 cm^−1^ and C=O vibration bands at 1770–1700 cm^−1^, corresponding to urethane bonds were observed. No NCO absorption band at 2270–2250 cm^−1^ has been observed confirming complete conversion of the isocyanate. Moreover, REF foams exhibited a higher intensity of the C–H vibration band of the methyl group at 2960–2875 cm^−1^, due to the presence of solely petrochemical polyol containing methyl groups of propylene oxide. Petrochemical polyol in modified foams was replaced in 30 wt% with PHOSO polyol, which in turn contains more methylene moieties, as confirmed by a larger band at 2960–2900 cm^−1^ coming from asymmetric C–H stretching vibrations of the methylene groups.

**Figure 5:**
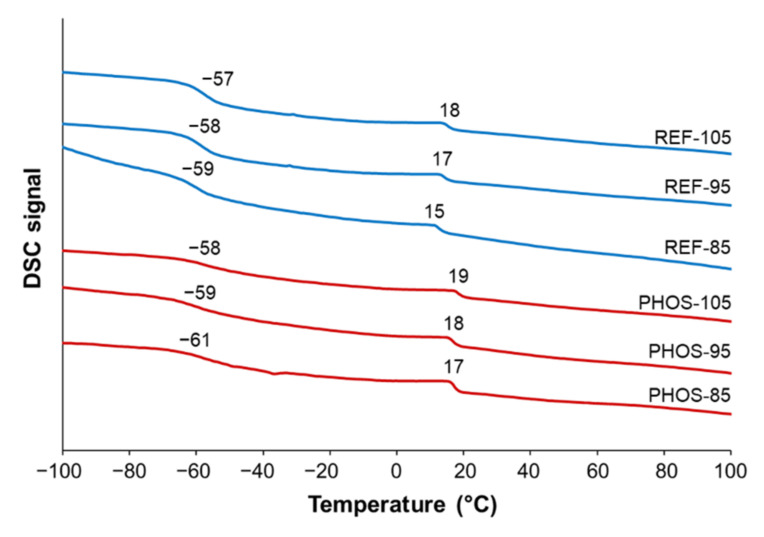
DSC curves of flexible foams (inert atmosphere).

**Figure 6:**
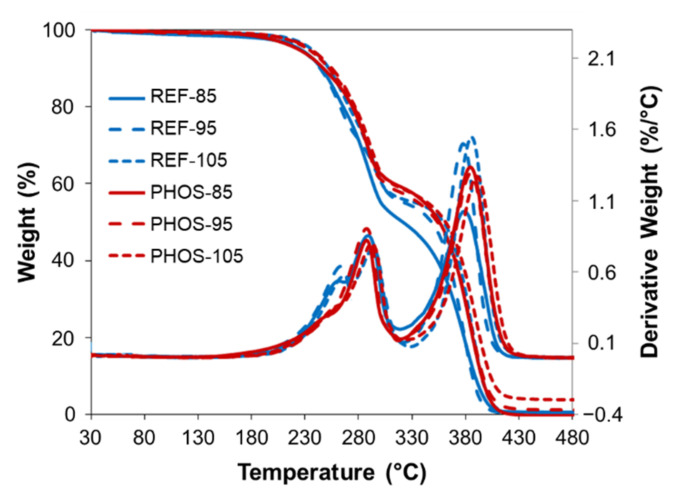
TGA and DTG curves of flexible foams (inert atmosphere).

Both DSC and TGA curves exhibited similar thermal behavior (Figure 5 and Figure 6). DSC curves showed two *T*_g_ values indicating the presence of two phases: soft phase (at lower *T*_g_ values) and rigid phase (at higher *T*_g_ values) in the polyurethane matrix. In polyurethanes, the phase-separated morphology is associated with reaction kinetics and determined by a complex system of reactions and phase evolution events [42]. When all reactants are mixed, water and isocyanate quickly react to form ureas and polyureas-based hard segments (HS), while polyol and isocyanate react at a slower rate to form urethanes and polyol-based soft segments (SS). At the beginning of polymerization, both HS and SS are soluble in the foaming mixture. As reactions proceed, a single-phased mixture transitions to a phase-separated system comprised of HS-rich hard domains and SS-rich soft domains. The process of phase separation and growth stops when the hard segments vitrify. The final morphology of the polyurethane foam is a result of the dynamics among reaction kinetics, phase separation, and vitrification. The introduction of crosslinker and vegetable oil-based polyol results in foams with higher HS concentrations compared to control ones. Slow-reacting bio-based polyol affects foam’s final morphology resulting in a high *T*_g_ oil-based polyol-rich phase and improved hard domain ordering. Whereas the low molecular weight crosslinker mixes into the hard domains, disrupts hard domain ordering, and alters interdomain spacing. Bio-based polyol, contrary to crosslinker, does not swell hard domains. The resulting polyurethane foam is phase-separated into domains rich in either polyurea segments or polyol segments [43,44]. The occurrence of these two phases ensures good properties of the polyurethane foams. The soft segments consist of polyol molecules and provide elasticity, whereas the hard segments are composed of diisocyanate moieties, and they contribute to the strength and rigidity through physical cross-linking points.

PHOSO and M5000 have different values of molecular weight, 1850 g/mol and 7150 g/mol, respectively. This difference has an impact on the crosslinking density of the foams. However, since bio-based foams were obtained by 30 wt% substitution of M5000 polyol with PHOSO, even if PHOSO has dangling chains having a plasticizing effect on the foams, still the crosslinking density of PHOS foams is higher than in the reference foams. In the case of flexible foams modified with PHOSO, *T*_g_ of soft phase tended to have lower values as the effect of plasticization of fatty acids’ dangling chains. *T*_g_ value of foam samples decreased e.g., from −59 °C (for reference foam REF-85) to −61 °C (for modified foam PHOS-85).

Two stages of degradation can be distinguished in the course of TGA and DTG curves of the foams (Figure 6). The first stage of degradation to the temperature of 291 °C comes from the dissociation of urethane and urea bonds in HS, while the second stage of degradation results from the breakdown of the ether bonds in SS. One can observe that the foams modified with bio-based polyol are slightly more thermally stable than reference foams. DTG curves show two peaks with the maxima around 280 °C and 380 °C. Table 3 summarizes temperatures of degradation and char values at 500 °C for the analyzed polyurethane foams.

In the case of flexible foams, it is important to determine their apparent density and mechanical properties (Table 4). Changes in the foaming process influence the apparent density of the foams. Typically, flexible polyurethane foams have an apparent density in the range 22–44 kg/m^3^. In the present study, apparent density decreased due to increased blowing agent production with the increase of isocyanate index. Moreover, the apparent density of the foams modified with PHOSO was slightly higher than in the case of reference foams. That can be related to the higher viscosity of PHOSO, than of replaced polyol M5000. Another reason can be the lower reactivity of PHOSO which has hydroxyl groups in the middle of the fatty acid chains, whereas M5000 contains highly reactive primary hydroxyl groups at the ends of polymeric chains.

**Figure 7:**
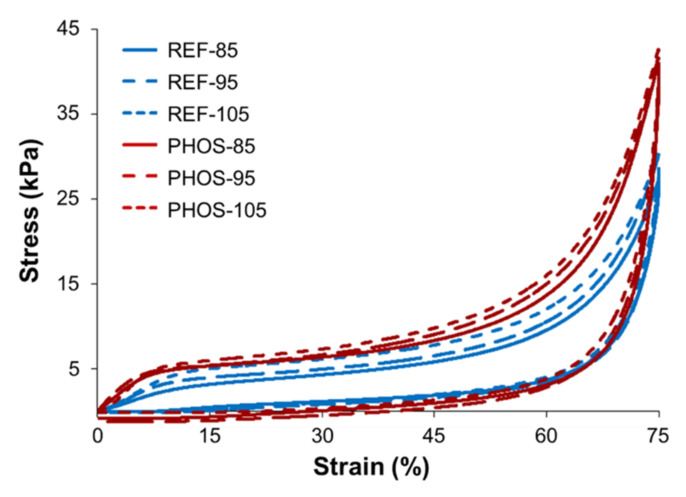
Hysteresis loops of flexible foams.

The loading and unloading stress-strain characteristics of flexible foams was investigated through hysteresis loops which determine the ability of foamed materials to absorb energy (Figure 7). The foams absorbed kinetic energy during the loading cycle and dissipated it as heat. The energy lost during deformation was measured as the difference between the energy used to load the sample and the energy used to unload the sample. The hysteresis loss was calculated as the ratio of energy lost during deformation (the area between the curves) to the energy used to load the sample. Its value allowed to determine the energy absorbing properties of the foamed materials. The lower the hysteresis loss, the better the foam behaves in terms of recovery of the energy absorbed during compression. On the other hand, higher hysteresis loss, as observed for PHOS foams, indicates that these foams are more energy absorbent and dissipating (desired in shock-absorbing materials). The hysteresis data observed for PHOS foams are consistent with their higher compression strength and lower resilience values in comparison to reference foams.

PHOS foams gave superior values of compression properties compared to unmodified foams. It is caused by the shorter SS, higher content of HS, and the occurrence of vegetable oil-based polyol dangling chains in the polyurethane matrix. M5000 has hydroxyl groups at the ends of polymeric chains, whereas PHOSO has the hydroxyl functionalities in the middle of the fatty acid chains. Therefore, M5000 has shorter and less flexible SS. The distance between the two hydroxyl groups in PHOSO is higher than in M5000. Moreover, higher *HV* of PHOSO (201.4 mg KOH/g) vs. M5000 (35.8 mg KOH/g) implies that more diisocyanate must be used to prepare the foams with PHOSO at the same isocyanate index as in the reference system. Higher hysteresis loss of PHOS foams can be caused also by the presence of free fatty acid chains.

Support factor is the ratio of the compressive stress at 65% compression (CLD_65_) to the compressive stress at 25% compression (CLD_25_). The higher the number, the better the ability of the foam to provide support, as the material is soft on the surface but provides greater support as compression increases. Flexible foams were characterized by the support factor with values ca. 3, defining the materials as comfortable. The support factor was slightly higher for the modified foams (PHOS-95 and PHOS-105 vs. REF-95 and REF-105, respectively).

Additionally, a ball rebound resilience test was performed to evaluate foams’ resilience by vertically dropping a steel ball on the foams from the required height. Based on the ball rebound test, flexible foams can be classified as high resilience foams (>50%), typical flexible foams (25–50%) or viscoelastic foams (<50%). Both REF foams and PHOS foams exhibited the values of resilience in the range 25–50% (corresponding to the typical flexible foams). An addition of PHOSO in the foams’ formulations resulted in lower resilience values. It was caused by the reduced elasticity of the foams resulted from a shorter distance between hydroxyl groups and a higher HS amount in the polyurethane matrix. Moreover, the lower resilience of bio-based foams was caused by the presence of SS with a different length and having different mobility, leading at the same time to better energy absorption.

**Figure 8:**
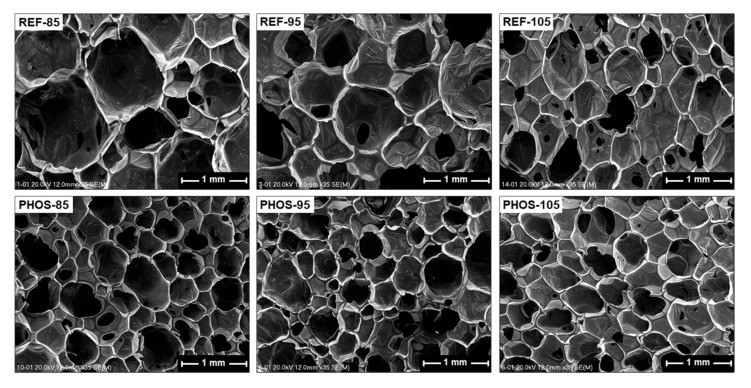
SEM micrographs of flexible foams.

The mechanical properties of foams are closely related to their apparent density values, but also the cell structure and chemical composition of the polyurethane matrix. This is mainly due to the differences in the ratio of the number of HS to the number of SS in the foams. All these factors influence the pore sizes and their distribution in the flexible foams, where bigger pores are observed for the reference foams while smaller and more evenly distributed pores are present in the bio-based foams. Figure 8 shows SEM micrographs of polyurethane foams obtained at 35× magnification in the perpendicular direction to the foam growth. One can observe that the cellular structure of the foams modified with bio-based polyol is more homogeneous and characterizes with a greater content of pores having a smaller size compared to the reference foams. Vegetable oil-based polyols support the action of the surfactant because they consist of hydrophilic ester groups and hydroxyl groups as well as hydrophobic chains of fatty acids.

## 4. Conclusions

Partial substitution of petrochemical polyol by vegetable oil-based polyol in the polyurethane foam formulations contributes to an increase in the share of renewable sources in the polyurethane matrix. Here, we presented new bio-based flexible polyurethane foams modified with polyols coming from the thiol-ene coupling of high oleic sunflower oil with 2-mercaptoethanol. Bio-based polyols affected the distribution of soft and hard segments in the polyurethane foams, which in turn determined their physico-mechanical properties.

The efficient thiol addition onto high oleic sunflower oil was demonstrated. Interestingly, the photoreaction was carried out under mild conditions, without using solvent and photoinitiator, and the product was easily purified. The reactions of intermolecular recombination occurred; however, despite these side reactions, byproducts contained hydroxyl groups. The synthesized high oleic sunflower oil-based polyol has been used as a partial (30 wt%) substituent of petrochemical polyether polyol in the formulations of flexible polyurethane foams. Obtained flexible foams with tailored properties can find broad applications as e.g., mattresses or automotive seats. The next step in the synthesis of flexible foams could be application of vegetable oils having higher iodine values ensuring the synthesis of polyols with different content and distribution of hydroxyl groups.

## Figures and Tables

**Table 1:** Formulations used to prepare flexible polyurethane foams.

Component		Formulation ID					
		REF-85	REF-95	REF-105	PHOS-85	PHOS-95	PHOS-105
Polyols	M5000	100	100	100	70	70	70
	PHOSO	–	–	–	30	30	30
Water		6.5	6.5	6.5	6.5	6.5	6.5
Surfactant	DABCO DC5950	3.1	3.1	3.1	3.1	3.1	3.1
Catalysts	DABCO NE300	0.14	0.14	0.14	0.14	0.14	0.14
	DABCO NE1070	0.18	0.18	0.18	0.27	0.27	0.27
	POLYCAT 15	0.07	0.07	0.07	0.07	0.07	0.07
	DABCO BL-11	0.12	0.12	0.12	0.12	0.12	0.12
	DABCO 33-LV	0.16	0.16	0.16	0.16	0.16	0.16
Isocyanate	TDI	61.1	68.3	75.5	68.0	76.2	84.0
NCO index		0.85	0.95	1.05	0.85	0.95	1.05

**Table 2:** Properties of HOSO and polyols: bio-based polyol PHOSO and petrochemical polyol M5000.

Substrate	$M_{n}$ (g/mol)	$M_{w}$ (g/mol)	*Ð*	AV (mg KOH/g)	HV (mg KOH/g)	$f_{OH}$	Water Content (wt%)	Viscosity at 25 °C (mPa·s)
HOSO	1290	1350	1.05	0.4	–	–	0.0244	36.6
PHOSO	1710	1850	1.08	0.4	201.4	6.14	0.7648	987.4
M5000	6760	7150	1.06	0.1	35.8	3.00	0.5249	844.0

**Table 3:** Temperatures of degradation and values of char at 500 °C of flexible foams.

**Sample**	$T_{d\;5\%}$ **(°C)**	$T_{d\;10\%}$ **(°C)**	$T_{d\;15\%}$ **(°C)**	$T_{d\;30\%}$ **(°C)**	$T_{d\;50\%}$ **(°C)**	$T_{d\;max}$ **(°C)**	Char at 500 °C (%)
REF-85	223	245	256	282	321	378	0
REF-95	225	244	255	284	348	379	0
REF-105	236	252	262	288	359	386	0
PHOS-85	225	247	263	288	321	385	0
PHOS-95	232	252	266	287	354	385	1
PHOS-105	234	255	267	291	361	389	4

**Table 4:** Apparent density and mechanical properties of flexible foams.

Parameter	Foam ID					
	REF-85	REF-95	REF-105	PHOS-85	PHOS-95	PHOS-105
Apparent density (kg/m^3^)	25.3 (±0.69)	24.6 (±0.74)	23.5 (±0.55)	26.3 (±0.79)	25.7 (±0.64)	24.4 (±0.51)
CLD_40_ (kPa)	5.2 (±0.09)	5.8 (±0.12)	7.1 (±0.17)	7.4 (±0.20)	8.0 (±0.24)	8.2 (±0.27)
CLD_65_ (kPa)	12.4 (±0.50)	13.6 (±0.70)	15.2 (±0.41)	17.9 (±0.55)	19.5 (±0.42)	20.0 (±0.46)
CLD_75_ (kPa)	27.1 (±0.40)	30.0 (±0.50)	30.3 (±0.55)	40.0 (±0.58)	41.0 (±0.46)	41.6 (±0.51)
Support factor	3.20 (±0.02)	2.98 (±0.02)	2.66 (±0.03)	3.01 (±0.04)	3.19 (±0.04)	3.25 (±0.04)
Hysteresis loss (%)	59.0 (±0.30)	66.7 (±0.43)	67.0 (±0.71)	78.0 (±0.93)	81.7 (±1.10)	87.4 (±1.52)
Resilience (%)						
parallel	49 (±0.43)	47 (±0.32)	45 (±0.38)	32 (±0.45)	30 (±0.36)	28 (±0.26)
perpendicular	50 (±0.34)	48 (±0.40)	46 (±0.41)	34 (±0.40)	32 (±0.25)	30 (±0.20)

Apparent density, CLD, Support factor, Hysteresis loss, Resilience = mean ± standard deviation of triplicate determinations.

## Data Availability

The data are available in a publicly accessible repository.

## Supplementary Materials

The following are available online at https://www.mdpi.com/article/10.3390/ma15020628/s1, Table S1: Fatty acid composition in HOSO, Figure S1: 1H NMR spectra of thiol-ene coupling of HOSO, Figure S2: FT-IR spectra of reference foam (REF-95) and foam modified with PHOSO polyol (PHOS-95).
