# Supplementary material for: Thiol-Ene Coupling of High Oleic Sunflower Oil towards Application in the Modification of Flexible Polyurethane Foams
Source: Materials (Basel). 2022 Jan 14;15(2):628. doi: 10.3390/ma15020628 (PMC8777839; doi:10.3390/ma15020628)
Supplement: Supplementary file 1 [file materials-15-00628-s001.zip › materials-1526423-supplementary.pdf]

materials

Supplementary Information

Table S1. Fatty acid composition in HOSO.

Fatty Acid		(mol%)
C14:0 [a]	myristic acid	0.1
C16:0	palmitic acid	4.1
C16:1 Δ 9c [b]	palmitoleic acid	0.06
C18:0	stearic acid	3.3
C18:1 Δ 9c	oleic acid	85.0
C18:2 Δ 9c, 12c	linoleic acid	6.0
C20:0	arachidic acid	0.3
C20:1 Δ 9c	gadoleic acid	0.3
C22:0	behenic acid	0.9

[a] C14:0 – number of carbon atoms : number of unsaturated double bonds.
[b] 9c – *cis* isomer of fatty acid (double bond at 9th carbon atom).

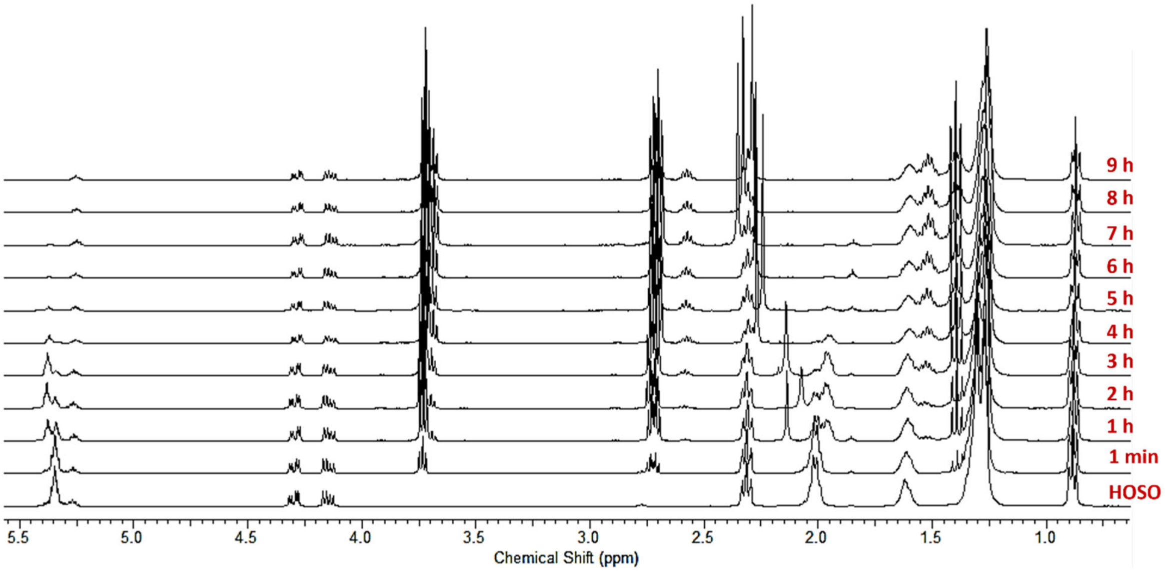

Figure S1. ^{1}H NMR spectra of thiol-ene coupling of HOSO.

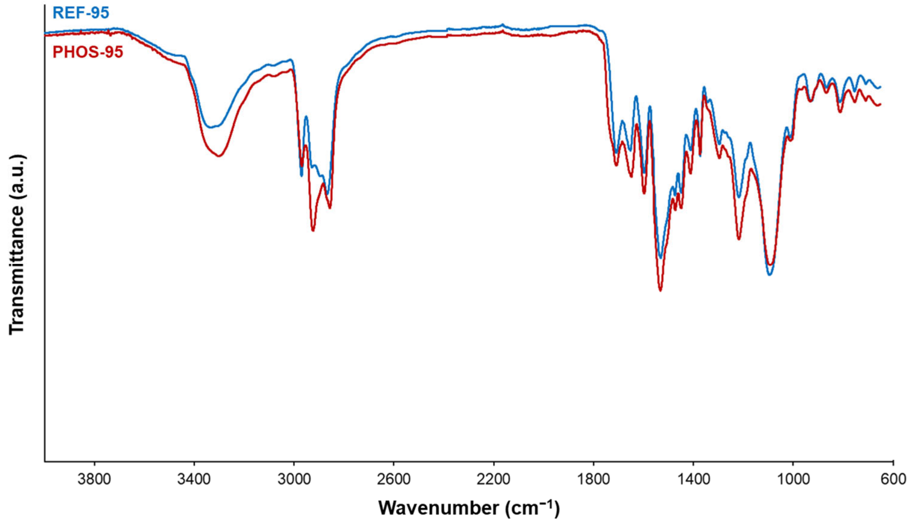

Figure S2. FT-IR spectra of reference foam (REF-95) and foam modified with PHOSO polyol (PHOS-95).
